# The Influence of HIV Status on Acute Appendicitis: A Retrospective Study from South Africa

**DOI:** 10.1007/s00268-023-07103-4

**Published:** 2023-08-14

**Authors:** Reza Laäs, Damian L. Clarke, Nicholas Dufourq, Michelle T. D. Smith, John L. Bruce, Mergan Naidoo

**Affiliations:** 1 Department of Emergency Medicine, Nelson R Mandela, School of Medicine, University of KwaZulu-Natal, 719 Umbilo Rd, Umbilo, Berea, 4001 South Africa; 2 Department of General Surgery and Trauma, Grey’s Hospital, 201 Townbush Road, Pietermaritzburg, 3201 South Africa; 3 Department of Emergency Medicine, Harry Gwala Regional Hospital, 89 Selby Msimang Road, Pietermaritzburg, 3201 South Africa; 4 Department of Anaesthetics and Critical Care, Grey’s Hospital, 201 Townbush Road, Pietermaritzburg, 3201 South Africa; 5 Discipline of Family Medicine, School of Nursing and Public Health, University of KwaZulu-Natal, Room 225F, George Campbell Building, King George V Avenue, Durban, South Africa

## Abstract

**Background:**

Despite the human immunodeficiency virus (HIV) being the most common comorbidity in South African surgical patients, its impact on appendicitis has not been well-described. We aimed to determine HIV status’ influence on patients’ presentation, assessment, management and outcomes with acute appendicitis.

**Methods:**

The retrospective chart review included all patients aged 12 years and older who were HIV-positive or HIV-negative and presented with acute appendicitis between 1 January 2013 and 31 December 2019. The primary outcome measure was survival to discharge. Secondary outcomes included analysis of the presentation (vital signs), assessment (biochemical, inflammatory markers) and management (intraoperative anatomical severity grading, length of hospital stay).

**Results:**

Of the 1096 patients with appendicitis, 196 (17.9%) were HIV-positive, and CD4 counts were available for 159. The median age was 23 years, with the HIV-positive patients being older and HIV-negative group having more males (58.7%). While the HIV-positive patients had a longer median length of hospital stay, there was no statistically significant difference in the two groups’ incidence of high-grade appendicitis (*p* = 0.670). The HIV-positive patients had a higher median shock index (OR 7.65; 95% [CI 2.042–28.64]) than their HIV-negative counterparts. HIV-positivity had a significant association with mortality (OR 9.56; 95% CI [1.68–179.39]), and of the seven HIV-positive patients who died, 66.7% (*n* = 4) had a CD4 < 200 cells/mm^3^ (OR 8.6; 95% CI [1.6–63.9]).

**Conclusion:**

HIV-positive patients, those with CD4 < 200 cells/mm^3^ or not on ART, have increased mortality risk and may benefit from increased perioperative surveillance. Patients with an unknown HIV status in a high-prevalence population should be offered HIV testing to risk stratify more accurately.

## Introduction

Appendicitis is the most common surgical emergency worldwide [1–3]. A systematic review of the global incidence of appendicitis in the twentieth century suggested a difference in incidence between low-, middle- and high-income countries, with the former reporting fewer cases than the latter [4]. This changed in the twenty-first century as low- and middle-income countries (LMIC), such as South Africa (SA), reported a rise in the incidence of appendicitis [1, 5]. Appendicitis in SA is associated with significant morbidity, as evidenced by threefold to fourfold higher appendiceal perforation rates than in high-income countries (HIC) [6]. SA has the largest population of people living with human immunodeficiency virus (HIV), with an estimated 7.97 million infected persons [7]. The province of KwaZulu-Natal (KZN) is disproportionately affected, with approximately 25% of adults being HIV-positive compared to a national average of 19% [7, 8].The HIV-positive population is growing due to increased access to antiretroviral therapy (ART) and prolonged life expectancy [9].

HIV is the most common comorbidity in the South African surgical population, [1, 10] and appendicitis is the most common surgical emergency [3, 11]. However, the correlation between HIV and appendicitis has not been well-described. While previous research suggests that HIV infection may be a risk factor for acute appendicitis, [3, 8, 12] inconsistencies have surfaced regarding the relationship between HIV, the clinical and biochemical presentation of appendicitis, and the associated morbidity and mortality [3, 13–17]. Current research suggests that HIV seropositivity is associated with delayed presentation, increased postoperative morbidity and a longer hospital stay [3, 15]. HIV infection exists along a spectrum of immuno-suppression, so this correlation may be necessary to identify patients at higher risk of morbidity and mortality. In this study, we investigated the relationship between HIV and appendicitis at Grey’s Hospital, a large tertiary referral public sector hospital in KwaZulu-Natal (KZN) Province. We aimed to determine the influence of HIV status on the presentation, assessment, management and outcomes of patients presenting with acute appendicitis.

## Material and methods

**Fig. 1:**
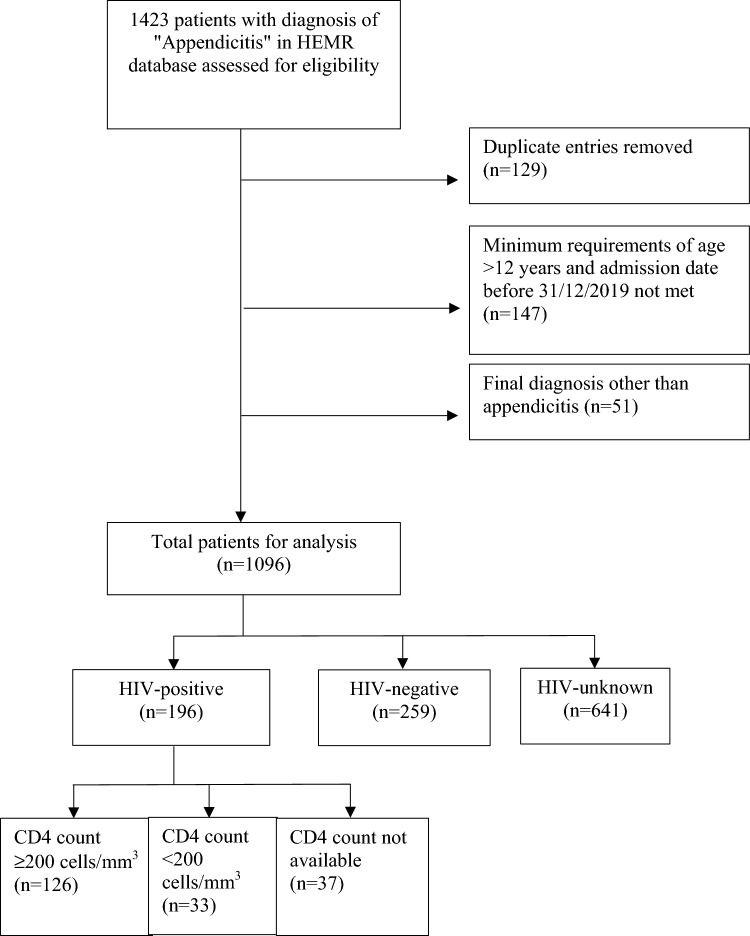
Consort diagram describing the total number of patients eligible for analysis and the excluded population. HIV, human immunodeficiency virus; *n*, number, CD4 expressed in cells/mm^3^

Grey’s hospital provides a tertiary surgical service to the western half of KZN with an estimated population of 4.5 million people. The surgery department has implemented a hybrid electronic medical record (HEMR) system, which facilitates data capture of all surgical patients. This was a retrospective, cross-sectional, analytical study that included all patients admitted with acute appendicitis between 1 January 2013 and 31 December 2019 who were 12 years of age or older. Patients were excluded if their final diagnosis was not acute appendicitis. Patients were classified as HIV-positive if they had a pre-existing diagnosis of HIV on admission or were diagnosed during admission. Patients were HIV-negative if documented as such on the database. Patients without an HIV status reported were ‘HIV-unknown’. HIV-unknown patients were excluded from the final analysis. (Fig. 1) Secondary confirmation of known HIV status was done using the National Health Laboratory Service (NHLS) database, from which the CD4 count and biochemical laboratory results were also collected. Patient records, which included the admission and discharge data, operative notes and morbidity records, were exported from HEMR as an excel spreadsheet. Data preparation was performed in Microsoft® Excel for Mac (version 16.59, One Microsoft Way, Redmond, Washington, USA). Data were analysed using R Studio (R Foundation for Statistical Computing for statistical analysis).

HIV-positive patients were compared to HIV-negative patients. Four main areas were assessed. These were physiological signs (temperature and shock index), biochemical markers (serum bicarbonate, base excess, urea and white cell count), anatomical severity grading using the American Association for the Surgery of Trauma (AAST) grading system for Emergency General Surgery (EGS) conditions and outcomes (survival to hospital discharge and length of hospital stay). A subgroup analysis was performed for HIV-positive patients with available CD4 counts. The patients were separated into a group with a CD4 count above and including 200 cells/mm^3^ and a group with counts below 200 cells/mm^3^.

Ethical approval was obtained from the UKZN BREC (BREC/00001381/2020), KwaZulu-Natal Department of Health Ethics Committee (NHRD Ref: KZ 202006 004), and Grey’s Hospital management before study commencement.

### Statistical analysis

Descriptive statistics were performed for the sample and subgroups. Continuous variables were described using medians and interquartile ranges (IQR) as these were non-normally distributed. Comparisons were then made using the Wilcoxon test. Categorical variables were expressed as frequencies and percentages and were compared using the Chi-square test or Fisher’s exact test where appropriate. When *p* values were < 0.05, odds ratios (OR) were calculated to confirm and quantify significance and were expressed with 95% confidence intervals. Univariate logistic regression was used to examine the relationships between independent variables and the ‘HIV positive’ dependent variable. Tukey’s transformation ladder was used to convert the variable’ length-of-stay (LOS)’ to a normal distribution. Linear regression was used to explore the relationship between LOS and HIV status, where LOS was the dependent variable. Univariate logistic regression was used to examine the relationships between the independent and dependent variables and the outcome of death. Multivariate logistic regression analysis was performed to define the relationship between patient characteristics and HIV status. No further model reduction was performed as only two independent predictors were identified.

## Results

Of the 1423 patients admitted to Grey’s Hospital with the diagnosis of appendicitis, 1096 were eligible for study inclusion, with high-grade appendicitis (AAST grade four or five) being observed in 427 (39%) patients. One hundred and ninety-six patients (17.9%) were HIV-positive, with CD4 counts available for 159 (81.1%). Two hundred and fifty-nine patients (23.6%) were documented HIV-negative. The HIV status was not recorded for 641 patients. Male patients accounted for 63.5% of the sample and had more than twice the odds of being unaware of their HIV status than female patients (OR 2.29; 95% CI [1.78–2.94]). (Table 1) All patients received antibiotics, with 1021 (93%) treated with Amoxicillin/Clavulanic acid. Hospital-acquired infections were escalated to second-line antibiotics, where Piperacillin/Tazobactam was administered (*n *= 7). Antibiotic treatment strategy did not differ if HIV-positive. All patients underwent operative management, with 756 patients (69%) undergoing midline laparotomies, 174 (15.9%) patients having Lanz incisions, and 166 (15.1%) patients undergoing laparoscopic appendicectomies. One hundred and seventy-four (15.9%) patients underwent a relaparotomy during their admission. Eighty-one patients required admission to the intensive care unit.

**Table 1:** Descriptive statistics of all patients with acute appendicitis presenting to Grey’s Hospital: predictors of mortality in overall group

	All *n *= 1096	Survived *n *= 1080	Died *n *= 16 (1.5%)	*p*	OR	95% CI
Age: median (IQR)	23 (17–33)	23 (17–33)	33 (28–52)	0.002	1.05	1.03–1.08*
Male	696 (63.5%)	688 (63.7%)	8 (50%)	0.35		
Rural	605 (55.25)	598 (55.4%)	7 (43.8%)	0.50		
HIV-positive	196 (17.9%)	189 (17.5%)	7 (43.8%)	0.007	3.7	1.30–10.0*
AAST grade 1–3	669 (61.0%)	664 (61.5%)	5 (0.5%)			
AAST grade > = 4	427 (39.0%)	416 (38.5%)	11 (68.8%)	0.028	3.5	1.3–11.2*
Shock index: median (IQR)	0.78 (0.66–0.94)	0.78 (0.66–0.93)	0.92 (0.72–1.0)	0.21		
Bic: median (IQR)	24.8 (22.5–26.5)	24.8 (22.6–26.5)	23.7 (20.6–26.4)	0.406		
BE: median (IQR)	0.4 (−1.8 to 2.6)	0.4 (−1.8 to 2.6)	−0.4 (−2.7 to 5.5)	0.828		
Urea: median (IQR)	4.1 (3.0–5.5)	4.1 (3.0–5.5)	5.2 (2.9–12.2)	0.356		
WCC: median (IQR)	12.9 (9.7–16.8)	12.9 (9.7–16.9)	10.8 (6.3–13.1)	0.061		
Temp: median (IQR)	36.8 (36.3–37.4)	36.8 (36.3–37.4)	36.6 (36.4–37.3)	0.879		

HIV, human immunodeficiency virus; AAST, The American Association for the Surgery of Trauma; SI, shock index; Bic, serum bicarbonate; BE, base excess; WCC, white cell count; Temp, temperature; OR, odds ratio; CI, confidence interval; IQR, interquartile ranges; *p*, *p*-value; *n*, number

^*^statistically significant odds ratio

### Comparison between the HIV-positive and HIV-negative patients

#### Demographic comparison

HIV-positive patients were older than the HIV-negative group (OR 1.04; 95% CI [1.03–1.04%]). The HIV-negative group had a higher proportion of males than the HIV-positive group (58.7% and 43.9%, respectively) (Table 2).

**Table 2:** Comparison between HIV-positive and HIV-negative patients presenting with acute appendicitis—Univariate analysis

	HIV-positive *n *= 196	HIV-negative *n *= 259	*p*	OR	95% CI
Age: median (IQR)	33 (26–40)	22 (16–32)	<0.001	1.04	1.03–1.06*
Male: median	86 (43.9%)	152 (58.7%)	0.002	0.55	0.38–0.80*
Rural	109 (55.6%)	134 (51.7%)	0.412		
Died	7 (3.6%)	1 (0.4%)	0.024	9.56	1.68–179.39*
LOS (days): median (IQR)	4 (2–7.3)	3 (2–5)	<0.001	1.17	1.10–1.25*
AAST grade > = 4	79 (40.3%)	84 (32.4%)	0.083		
SI: median (IQR)	0.81 (0.70–0.97)	0.76 (0.65–0.88)	0.001	5.84	2.29–15.48*
Bic: median (IQR)	24.4 (22.2–26.0)	24.7 (22.4–26.3)	0.236		
BE: median (IQR)	0 (−1.9 to 1.7)	0.6 (−1.9 to 2.6)	0.395		
Urea: median (IQR)	3.7 (2.7–5.2)	4.1 (3.0–5.4)	0.284		
WCC: median (IQR)	11.8 (8.3–15.3)	13.1 (9.7–16.6)	0.012	0.97	0.94–1.00
Temp: median (IQR)	36.8 (36.2–37.4)	36.6 (36.2–37.1)	0.055		

HIV, human immunodeficiency virus; AAST, The American Association for the Surgery of Trauma; SI, shock index; Bic, serum bicarbonate; BE, base excess; WCC, white cell count; Temp, temperature; OR, odds ratio; CI, confidence interval; IQR, interquartile ranges; *p*, *p*-value; *n*, number

*statistically significant odds ratio

#### Clinical and biochemical assessment

The median white cell count (WCC) was lower on admission for HIV-positive patients, although this disparity was not statistically significant (OR 0.97; 95% CI [0.94–1.00]) (Table 2). During multivariate logistic regression analysis, only age and shock index [OR 7.65; 95% CI 2.042–28.64] were found to be independent predictors of HIV-status (Table 3).

**Table 3:** Comparison between HIV-positive and HIV-negative patients presenting with acute appendicitis—multivariate analysis

	OR	95% CI	*p*
Age	1.059	1.03–1.08*	<0.001
Male	0.69	0.4–1.2	0.191
Rural	1.7	0.99–2.89	0.53
AAST grade > = 4	0.82	0.46–1.45	0.495
SI	7.65	2.042–28.64*	0.003
Bic	0.99	0.975–1.007	0.261
BE	1.02	0.97–1.07	0.457

HIV, human immunodeficiency virus; AAST, The American Association for the Surgery of Trauma; SI, shock index; Bic, serum bicarbonate; BE, base excess; OR, odds ratio; CI, confidence interval; *p*, *p*-value

*statistically significant odds ratio

#### Anatomical severity

The rate of high-grade appendicitis found at index laparotomy was 40.3% in the HIV-positive group and 32.4% in the HIV-negative group. This difference was not statistically significant (*p *= 0.083). (Table 2) Subgroup analysis of HIV-positive patients with known CD4 count indicated no correlation between CD4 < 200 cells/mm^3^ and high-grade appendicitis (AAST > = 4) (*p *= 0.407).

#### Outcomes

HIV-positive patients had a mortality rate of 3.6% (*n *= 7), compared to 0.4% (*n *= 1) in the HIV-negative group (Table 2). HIV-positive status was significantly associated with mortality (OR 9.56; 95% CI [1.68–179.39]). HIV-positive patients had a longer median length of hospital stay than the HIV-negative cohort (OR 1.17; 95% CI [1.10–1.25]). The findings of this primary outcome prompted further analysis to identify any predictors of mortality.

#### Predictors of mortality in the overall group

Those who died had a median age a decade older than those who survived (33 years and 23 years, respectively; OR 1.05; 95% CI [1.03–1.09]). HIV-positive patients were at greater odds of dying than those who were not (OR 3.7; 95% CI [1.3–10.0]). High anatomical severity of disease was also associated with increased mortality (OR 3.5; 95% CI [1.3–11.2]) (Table 1). The cause of death was sepsis with multiorgan failure in 14 patients (87.5%) (Table 4).

**Table 4:** Patient characteristics and cause of death among mortalities

Age (years)	Sex	HIV status	CD4 (cells/mm^3^)	AAST grading	Cause of death
14	Male	Positive	3	5	Sepsis
44	Male	Positive	5	1	Sepsis
30	Male	Positive	14	1	Sepsis
34	Female	Positive	138	1	Sepsis
32	Male	Positive	464	1	Sepsis
50	Male	Positive	1227	4	Sepsis
27	Female	Positive	–	5	Sepsis
34	Male	Negative	–	5	Sepsis
18	Female	Unknown	–	5	Sepsis
28	Male	Unknown	–	4	Sepsis
22	Female	Unknown	–	5	Sepsis
95	Female	Unknown	–	5	Sepsis
59	Female	Unknown	–	5	Sepsis
60	Female	Unknown	–	1	Renal failure
82	Male	Unknown	–	5	Appendiceal adenocarcinoma, multiorgan failure
30	Female	Unknown	–	4	Sepsis

HIV, human immunodeficiency virus; AAST, The American Association for the Surgery of Trauma

#### Predictors of mortality in the HIV-positive group

**Fig. 2:**
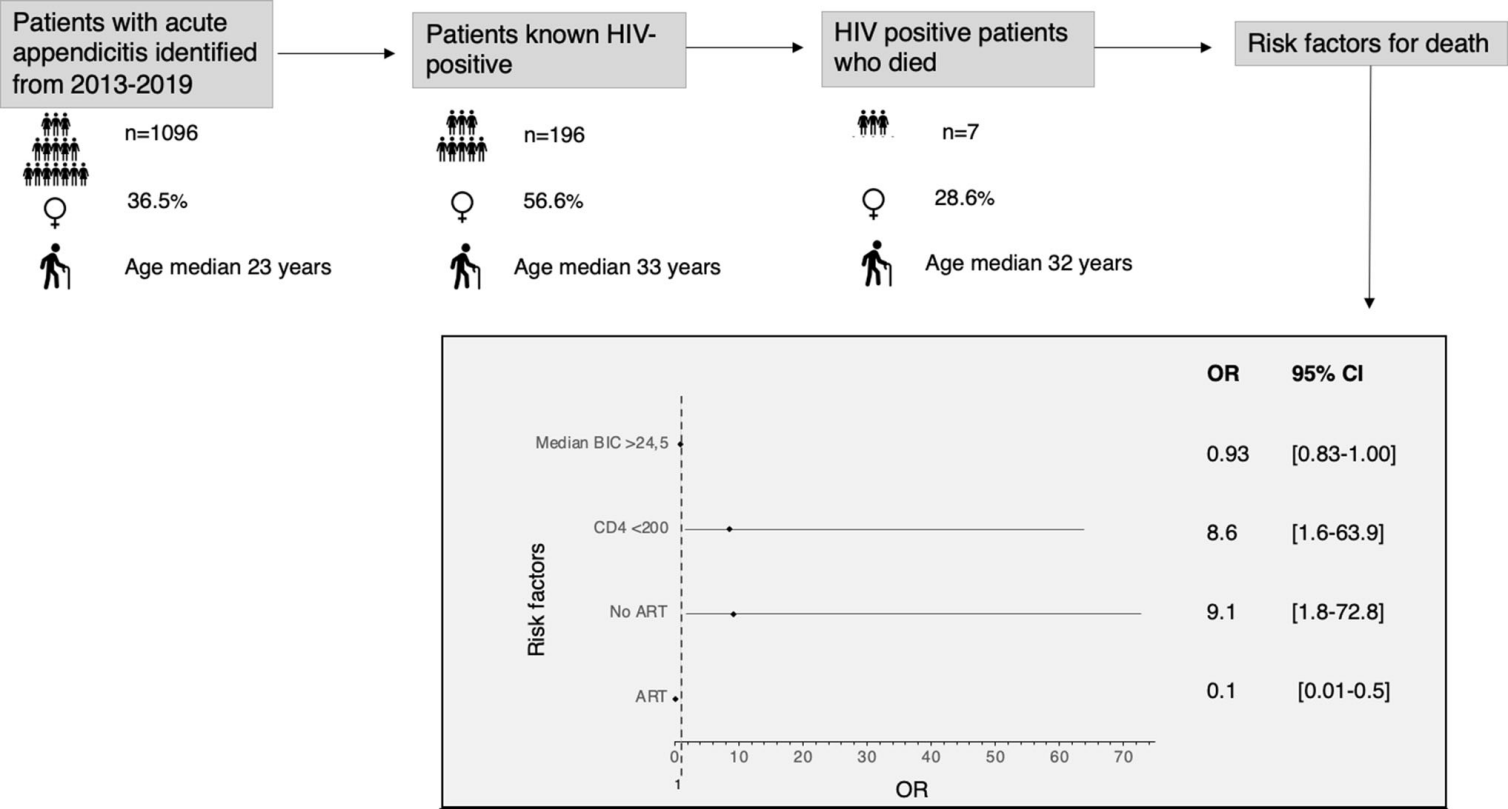
Summary illustration of main funding and forest plot indicating risk factors for mortality in the HIV-positive subgroup (Univariate analysis). HIV, human immunodeficiency virus; ART, antiretroviral therapy; Bic, serum bicarbonate; OR, odds ratio; CI, confidence interval; *n*, number,CD4 expressed in cells/mm^3^

CD4 count results were available for 159 of the 196 known HIV-positive patients, with the median CD4 count of those who died being 76 cells/mm^3^. One patient who died did not have a CD4 count available. (Table 5) Multivariate analysis was not done due to the low number of deaths. Summary of findings is included in Fig. 2.

**Table 5:** Predictors of mortality in HIV-positive patients with acute appendicitis

	Survived *n *= 189	Died *n *= 7	*p*	OR	95% CI
Age: median (IQR)	33 (28.5–39)	32 (28.5–39)	0.995		
ART	150 (79.4%)	2 (28.6%)	0.007	0.10	0.01–0.50*
No ART	39 (20.6%)	5 (71.4%)	0.007	9.1	1.8–72.8*
CD4: median (IQR) (*n *= 159)**	458 (250–638)	76 (7–383)	0.055		
CD4 < 200 cells/mm^3^ (*n *= 159)**	29 (18.2%)	4 (66.7%)	0.017	8.6	1.6–63.9*
Male	81 (42.9%)	5 (71.4%)	0.156		
Rural	107(56.6%)	2 (28.6%)	0.244		
AAST grade > = 4	76 (40.2%)	3 (42.9%)	1.0		
Shock Index: median (IQR)	0.81 (0.71–0.97)	0.77 (0.67–0.90)	0.528		
Bic: median (IQR)	24.5 (22.4–26.1)	20.6 (18.7–22.2)	0.030	0.93	0.83–1.00
BE: median (IQR)	0.1 (−1.9 to 1.8)	−1.8 (−5.7 to 0.4)	0.290		
Urea: median (IQR)	3.7 (2.7–5.2)	4.1 (2.2–5.5)	0.841		
WCC: median (IQR)	11.8 (8.4–15.5)	6.4 (3.8–12.7)	0.066		
Temp: median (IQR)	36.9 (36.2–37.4)	36.5 (36.2–37.0)	0.438		

CD4 expressed in cells/mm^3^

HIV, human immunodeficiency virus; ART, antiretroviral therapy; AAST, The American Association for the Surgery of Trauma; SI, shock index; Bic, serum bicarbonate; BE, base excess; WCC, white cell count; Temp, temperature; OR, odds ratio; CI, confidence interval; IQR, interquartile ranges; *p*, *p*-value; *n*, number

*statistically significant odds ratio

**Thirty-seven patients missing CD4 counts. Calculations performed on a sample of 159 (153 survived and 6 died)

## Discussion

HIV is the most common comorbidity among emergency general surgery patients undergoing laparotomy in the KZN public health sector. The impact of HIV in the context of surgical illness is poorly understood [18–21]. Initial reports of emergency surgery in patients with AIDS (acquired immunodeficiency syndrome) in the 1990s indicated high mortality rates of up to 70% [21]. However, since the advent and availability of ART in the early 2000s, emerging evidence suggests comparable mortality rates between HIV-positive and negative patients [19, 22, 23]. Our study found differences in the presentation and outcomes of patients with acute appendicitis in the context of HIV, with those being positive having a more extended hospital stay and increased mortality risk. The subgroup analysis of HIV-positive patients indicated that a CD4 < 200 cells/mm^3^ and ART naivety or non-compliance were risk factors for mortality in patients with acute appendicitis.

### Presentation

The HIV-positive patients were likely to be older (median age 33 years vs. 22 years), similar to other sub-Saharan countries [15, 16, 24]. HIV-positive patients in the USA also tended to be older than HIV-negative patients (42.8 vs. 33.6 years) [25]. The HIV-positive population in our study was predominantly female (56.1%). While Southern African research has shown similar findings [16, 26], the USA and Tanzania have indicated a male predominance [24, 25]. This study’s results may reflect the female predominance in the HIV-positive population in South Africa rather than a risk factor for appendicitis which is supported by male sex not being significant in the multivariate analysis. StatsSA 2019 indicated that over a fifth of females in South Africa aged 15–49 were HIV-positive [7]. Female patients are also more likely to be aware of their HIV status due to better health-seeking behaviour and primary healthcare strategies, resulting in the increased screening of females of reproductive age.

### Clinical presentation and biomarkers

In contrast to previous evidence, we did not find a statistically significant difference in WCC levels between HIV-positive and negative subgroups [3, 24–27]. This was also not apparent in the subgroup analysis of patients with a CD4 count lower than 200 cells/mm^3^. The shock index has not been described or validated in previous studies of HIV-positive patients with acute appendicitis. Our multivariate analysis indicated that HIV-positive patients are present with a higher shock index (0, 81) than their counterparts. A comparable parameter from previous research is the pulse rate, with two studies showing that HIV-positive patients presented with an increased pulse rate of greater than 100 [16, 26].

### Anatomical severity

South African studies in similar settings found no significant difference in patient presentation and incidence of surgical sepsis in patients with and without HIV [2, 8, 18]. AAST grading for EGS was introduced to measure the anatomical severity of the disease and has been broadly validated in multiple studies [28, 29]. Increasing AAST grading is associated with poorer outcomes, and grade-for-grade patients in South Africa are found to have worse outcomes [28]. In our study, high-grade appendicitis (AAST 4 or 5) was associated with increased mortality, although this was not shown to be influenced by HIV status or CD4 count. This finding was in keeping with international and local literature, where intra-operative severity scoring or perforation rates are not found to be increased in HIV-positive patients [3, 15, 16, 25, 26]. Therefore, despite not having severe AAST grading, HIV-positive patients had increased mortality, suggesting surgical findings of high-grade appendicitis cannot be relied upon to indicate mortality risk in the HIV-positive group.

### Outcome

Green et al. [8] found that a CD4 count below 200 cells/mm^3^ was associated with a significantly higher mortality rate of 60% in patients with surgical sepsis. A Tanzanian study on secondary peritonitis also indicated HIV infection significantly increased mortality [30]. While HICs have appreciably lower mortality rates for acute appendicitis than LMICs, a US study [25] showed that patients with HIV/AIDS had four times the mortality rate. Mortality in the general patient population increased in older patients (median age 33 years), AAST grades four or more, and those who were HIV-positive (3, 6%). HIV-induced immunosuppression is likely the underlying reason for increased mortality despite no increase in the AAST grading of appendicitis. The findings support that patients who are ART naïve or have CD4 cell counts less than 200 cells/mm^3^ have increased mortality.

The economic impact of acute appendicitis in HIV-positive patients has also been highlighted, as they are likely to have a longer hospital stay (4 days vs. 3 days). This is in keeping with other publications, which consistently show that HIV-positive patients are admitted for extended periods in both LMIC and HICs [15, 17, 24, 25]. Globalization and the success of ART may lead to increased HIV prevalence in HIC. It is, therefore, imperative to understand how HIV interacts with one of the most common surgical emergencies in the world.

We recommend that patients with the following risk factors may benefit from enhanced perioperative surveillance and monitoring:

Patient risk factors:

1. HIV-positive.
2. ART naïve or lost to follow-up.

Immunological risk factors:

(3) CD4 count < 200 cells/mm^3^

Intra-operative risk factors:

(4) AAST grade 4 or greater (general population)

We recommend that all surgical patients from a high HIV prevalence population presenting to a healthcare facility with unknown HIV status be offered testing. This would assist in identifying patients at risk and may benefit from increased perioperative surveillance.

## Limitations

Standard limitations and confounders of retrospective database analysis apply to our research. Socioeconomic factors may contribute to treatment delay and differences in outcomes, although this was not evident in patients from rural referral centres with similar mortality rates and HIV prevalence. A specific limitation is the sizeable HIV-unknown group and missing CD4 counts. Only 17% of our study population was HIV-positive, compared to a general population rate of 19% in South Africa and approximately 25% in KZN. This confounder points to many potentially HIV-positive (but undiagnosed) patients in the HIV-unknown cohort. This limitation is subject to record keeping and lack of HIV testing performed or documented on the HEMR database. To decrease inaccuracy, only patients with confirmed HIV and CD4 results documented were included and secondary confirmation was done using the NHLS database. Due to the retrospective nature of the study design, the missing data were unavoidable.

## Conclusion

To ameliorate the risk of morbidity and mortality, our findings indicate that HIV-positive patients with CD4 cell counts less than 200 cells/mm^3^ and are ART naïve would benefit from increased perioperative surveillance. Patients with an unknown HIV status in a high-prevalence population should be offered HIV testing to assist in risk stratification and ART initiation. Strategies to alleviate risk in the HIV-positive patient provide an avenue for future study.
